# ALWPs Improve Cognitive Function and Regulate Aβ Plaque and Tau Hyperphosphorylation in a Mouse Model of Alzheimer’s Disease

**DOI:** 10.3389/fnmol.2019.00192

**Published:** 2019-08-16

**Authors:** Youngpyo Nam, Bitna Joo, Ju-Young Lee, Kyung-Min Han, Ka-Young Ryu, Young Ho Koh, Jeongyeon Kim, Ja Wook Koo, Young-Man We, Hyang-Sook Hoe

**Affiliations:** ^1^Department of Neural Development and Disease, Korea Brain Research Institute (KBRI), Daegu, South Korea; ^2^Department of Brain & Cognitive Sciences, Daegu Gyeongbuk Institute of Science & Technology (DGIST), Daegu, South Korea; ^3^Center for Biomedical Sciences, Center for Infectious Diseases, Division of Brain Disease, Korea National Institute of Health, Heungdeok-gu, South Korea; ^4^College of Korean Medicine, Wonkwang University, Iksan, South Korea

**Keywords:** Aβ, tau, Alzheimer’s disease, amyloid plaque, long-term memory, dendritic spines

## Abstract

Recently, we reported that ALWPs, which we developed by combining Liuwei Dihuang pills (LWPs) with antler, regulate the LPS-induced neuroinflammatory response and rescue LPS-induced short- and long-term memory impairment in wild-type (WT) mice. In the present study, we examined the effects of ALWPs on Alzheimer’s disease (AD) pathology and cognitive function in WT mice as well as 5x FAD mice (a mouse model of AD). We found that administration of ALWPs significantly reduced amyloid plaque levels in 5x FAD mice and significantly decreased amyloid β (Aβ) levels in amyloid precursor protein (APP)-overexpressing H4 cells. In addition, ALWPs administration significantly suppressed tau hyperphosphorylation in 5x FAD mice. Oral administration of ALWPs significantly improved long-term memory in scopolamine (SCO)-injected WT mice and 5x FAD mice by altering dendritic spine density. Importantly, ALWPs promoted spinogenesis in primary hippocampal neurons and WT mice and modulated the dendritic spine number in an extracellular signal-regulated kinase (ERK)-dependent manner. Taken together, our results suggest that ALWPs are a candidate therapeutic drug for AD that can modulate amyloid plaque load, tau phosphorylation, and synaptic/cognitive function.

## Introduction

Alzheimer’s disease (AD) is an age-related neurodegenerative disease (Deary and Whalley, 1988; Hardy and Selkoe, 2002) with several major pathological hallmarks reflecting the disease mechanism. Recent clinical evidence strongly supports the involvement of amyloid β (Aβ) and tau in AD (Deary and Whalley, 1988; Lee et al., 2010). Specifically, abnormal hyperphosphorylation of tau and accumulation of Aβ plaques can lead to dendritic spine loss, synaptic dysfunction, neuronal cell death, and memory impairment (Hardy and Selkoe, 2002). For instance, several studies have demonstrated that soluble Aβ isolated from AD brains or Aβ oligomer impairs long-term potentiation (LTP) and increases long-term depression (LTD) through an α-amino-3-hydroxy-5-methyl-4-isoxazolepropionics acid (AMPA) receptor-dependent signaling pathway (Li et al., 2013; Reinders et al., 2016). In addition, aggregation of tau causes dendritic spine loss and impairment of synaptic function by inducing the disassembly of microtubules (Thies and Mandelkow, 2007; Nisbet et al., 2015). Thus, both Aβ and tau appear to be involved in dendritic spine loss and synaptic dysfunction, which lead to cognitive decline in AD, and drugs that can reduce both Aβ plaques and tau phosphorylation are therefore therapeutic candidates for AD.

Liuwei Dihuang pills (LWPs) are a traditional oriental herbal medicine for the treatment of diabetes mellitus and malfunctions of the immune system (Park et al., 2005; Lee et al., 2012). LWPs contain six different herbal components: steamed *Rehmanniae radix*, *Discoreae radix*, *Corni fructus*, *Hoelen*, *Moutan* *cortex radices*, and *Alismatis radix* (Sangha et al., 2012). Several studies have demonstrated that LWPs affect learning and memory in D-galactose-induced aging and ibotenic acid-induced amnesia rodent models (Kang et al., 2006; Zhang et al., 2011). However, whether LWPs can modulate AD pathology and synaptic function has not been examined in detail.

Antler is widely used as a traditional oriental medicine and affects several biological functions. For instance, molecules secreted from antlers can facilitate neurite outgrowth and axonal growth (Gray et al., 1992; Li et al., 2007; Pita-Thomas et al., 2010). In addition, antler can regulate neuroinflammatory responses and cognitive performance (Lee et al., 2010; Dong et al., 2018). Based on the literature on LWPs and antler, we hypothesized that ALWPs containing antler and LWPs might have synergistic effects on LPS-induced neuroinflammation and LPS-induced memory impairment. Indeed, we recently demonstrated that ALWPs have additive effects on LPS-mediated neuroinflammatory responses compared with the individual components of ALWPs (Lee et al., 2018). In addition, we found that oral administration of ALWPs improved short-term and long-term memory in LPS-injected wild-type (WT) mice (Lee et al., 2018).

In the present study, we further examined whether ALWPs can affect AD pathology (including Aβ plaque and tau hyperphosphorylation) and found that oral administration of ALWPs significantly decreased amyloid plaque number as well as tau hyperphosphorylation in the cortex and hippocampus of 5x FAD mice, a model of AD. In addition, oral administration of ALWPs to scopolamine (SCO)-injected WT mice and 5x FAD mice rescued deficits in long-term memory and promoted dendritic spine number. ALWPs treatment promoted dendritic spine formation in both primary hippocampal neurons and WT mice. Importantly, ALWPs increased dendritic spine number in an extracellular signal-regulated kinase (ERK)-dependent manner in primary hippocampal neurons. Taken together, these data suggest that ALWPs may be a useful potential drug for preventing and/or treating AD.

## Materials and Methods

### Ethics Statement

All experiments were approved by the institutional biosafety committee (IBC) of the Korea Brain Research Institute (KBRI, approval no. 2014-479).

### Cell Lines and Culture Conditions

COS7 (monkey kidney) cells were maintained in DMEM-high glucose (HyClone, USA) supplemented with 10% fetal bovine serum (FBS, HyClone, USA) in a 5% CO_2_ incubator. Amyloid precursor protein (APP)-H4 cells (H4 cells overexpressing human APP and producing high levels of Aβ were maintained in DMEM-high glucose supplemented with 10% FBS and gentamycin in a 5% CO_2_ incubator.

### Wild-Type Mice

**Figure 1:**
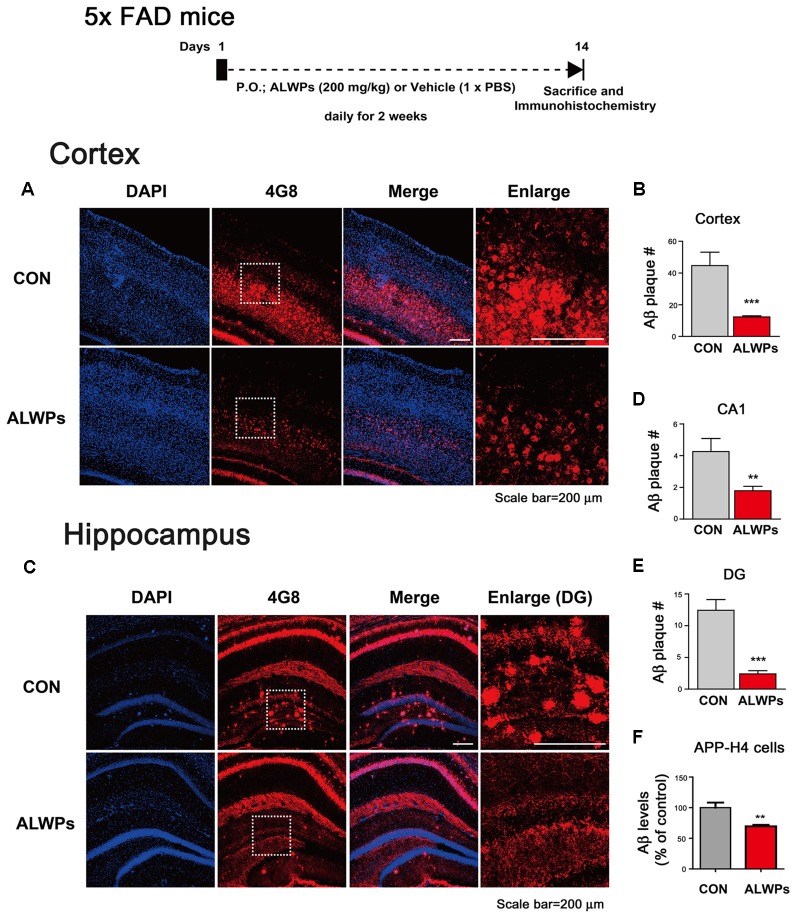
ALWPs significantly decrease amyloid plaque levels in 5x FAD mice. **(A)** 5x FAD mice were orally administered ALWPs (200 mg/kg, p.o.) or PBS daily for 2 weeks, followed by immunostaining of brain slices with an anti-4G8 antibody. Representative images of the cortex are shown. **(B)** Quantification of data from (**A**; con, *n* = 6 mice; ALWPs, *n* = 6 mice). **(C)** Representative images of the hippocampus of 5x FAD mice administered ALWPs (200 mg/kg, p. o.) or PBS daily for 2 weeks. **(D,E)** Quantification of data from (**C**; CA1 and DG; con, *n* = 6 mice; ALWPs, *n* = 6 mice). **(F)** Amyloid precursor protein (APP)-H4 cells (H4 cells overexpressing human APP and producing high levels of Aβ were treated with ALWPs (500 μg/ml) or PBS for 24 h, and human Aβ ELISA was performed (con, *n* = 22; ALWPs, *n* = 22). **p < 0.01, ***p < 0.0001.

All procedures were approved by the Institutional Animal Care and Use Committee (IACUC-2016-0013) of KBRI. Adult WT C57BL6/J male mice (8 weeks old, 25–30 g; Orient-Bio Company, Gyeonggi-do, South Korea) were used in the experiments. The animals were housed under a 12-h light/dark cycle with food and water *ad libitum* in a pathogen-free facility. For all experiments, mice were randomly assigned to the control (PBS) or treatment (ALWPs) group. We used 40 or 37 mice for each of the Y-maze and novel object recognition (NOR) tests. After adaptation for 1 week, the mice were randomly divided into three groups: Y-maze: (1) control (PBS, *n* = 13); (2) scopolamine (SCO, 1 mg/kg; *n* = 13); and (3) ALWPs (200 mg/kg) + SCO (1 mg/kg; *n* = 14); NOR: (1) control (PBS, *n* = 12); (2) SCO (1 mg/kg; *n* = 12); and (3) ALWPs (200 mg/kg) + SCO (1 mg/kg; *n* = 13). The doses of ALWPs were determined based on our previous report (Lee et al., 2018). The mice were orally administered PBS or ALWPs (200 mg/kg) daily for 11 days (days 1–11). Beginning on day 3, the mice were injected with SCO (1 mg/kg) daily for 9 days (days 3–11), and the Y-maze test was conducted on day 10. On day 11, training sessions for NOR were conducted. After NOR training, SCO (1 mg/kg) was injected, and the NOR test was performed on the following day. Mice that showed a low interaction time (<7 s in NOR training were regarded as insufficiently trained and eliminated from further testing. The *in* *vivo* experimental design is summarized in Figure 1.

### 5x FAD Mice

All animal experiments were performed in accordance with approved animal protocols and guidelines established by KBRI (IACUC-2016-0013). 5x FAD transgenic (Tg) mice (stock number 008730, B6SJL-Tg APPSwFlLon, PSEN1*M146L*L286V6799Vas/Mmjax) were purchased from Jackson Laboratory, and only male mice were used for this study. For the behavior experiments, 5x FAD mice were orally administered PBS or ALWPs (200 mg/kg) daily for 14 days, and Y maze and NOR tests were conducted. The mice were randomly divided into two groups: Y-maze: (1) control (PBS, *n* = 8); and (2) ALWPs (200 mg/kg; *n* = 8); NOR: (1) control (PBS, *n* = 8); and (2) ALWPs (200 mg/kg; *n* = 8). After the behavior experiments, we conducted Golgi staining to measure dendritic spine density (*n* = 8 mice/group). To examine the effects of ALWPs on Aβ plaque levels, 5x FAD mice were orally administered PBS or ALWPs (200 mg/kg, p.o.) daily for 2 weeks, and immunohistochemistry was conducted with an anti-4G8 antibody (*n* = 6 mice/group). In addition, 5x FAD mice were orally administered PBS or ALWPs (200 mg/kg, p.o.) daily for 2 weeks, and immunohistochemistry was conducted with various anti-tau antibodies to determine the effects of ALWPs on tau phosphorylation (*n* = 3 mice/group). Data were analyzed in a semi-automated manner using ImageJ software, and the results were confirmed by an independent researcher who did not participate in the current experiments.

### Y-Maze and NOR Tests

Y-maze and NOR tests were performed to assess cognitive function as described previously (Lee et al., 2018). All experiments were performed under low red light (4–5 lux). For the Y-maze test, mice were placed in a Y-shaped maze with three arms at 120° angles (36.5 cm × 7 cm × 15.5 cm). The mice were allowed to explore for 3 min, and the number of arms visited and their sequences were recorded using SMART 3.0 software (Panlab Harvard Apparatus). The percentage of alternation (%) was calculated using the following formula: number of alternation triplets/(total number of arm entries − 2) × 100. For the NOR test, mice were placed in an experimental apparatus (30 cm × 30 cm × 25 cm) containing two identical objects for training. They were allowed to freely move to explore the objects for 3 min. On the following day, the mice were placed in the same apparatus with two objects for 5 min. One was a familiar object, which was the same object experienced previously, and the other was a novel object, which was a new item that the mice had never experienced before. All trials were video-recorded, and the interaction preference (%) was calculated using the following formula: interaction time with the novel object/(total interaction time with two objects) × 100.

### Antibodies

We used the following primary antibodies for this study: mouse anti-4G8 (1:1,000, BioLegend, San Diego, CA, USA), mouse anti-6E10 (1:1,000, BioLegend, San Diego, CA, USA), rabbit anti-APP N-terminus (1:1,000, Sigma, Ronkonkoma, NY, USA), rabbit anti-ADAM8 (1:200, LSBio, Seattle, WA, USA), rabbit anti-ADAM9 (1:200, Abcam, UK), rabbit anti-ADAM10 (1:200, Abcam, UK), rabbit anti-ADAM12 (1:200, Abcam, UK), rabbit anti-ADAM17 (1:200, Abcam, UK), mouse anti-BACE1 (1:200, Abcam, UK), rabbit anti-neprilysin (1:200, Millipore, Burlington, MA, USA), mouse anti-AT100 (1:500, Invitrogen, Carlsbad, CA, USA), mouse anti-AT180 (1:500, Invitrogen, Carlsbad, CA, USA), and mouse anti-Tau5 (1:500, Invitrogen, Carlsbad, CA, USA).

### Preparation of ALWPs

ALWPs included LWPs (Yukmijuhwang-tang: *Rehmannia glutinosa*, *Cornus officinalis*, *Discoreae rhizoma, Paeonia suffruticosa*, *Poria cocos*, and *Alisma orientale*), *Lycium chinense*, *Polygala tenuifolia*, *Acorus gramineus*, and *Antler*. ALWPs were prepared through a three-step extraction process. First, *Lycium chinense* (1,000 g), *Rehmannia glutinosa* (1,200 g), *Dioscorea rhizoma* (500 g), *Cornus officinalis* (500 g), *Paeonia suffruticosa* (360 g), *Alisma orientale* (360 g), *Poria cocos* (360 g), and *Antler* (240 g) were boiled for 12 h at 120°C. Next, the heated mixture was sifted through a filter to remove debris. *Polygala tenuifolia* (180 g), *Acorus gramineus* (180 g), and *Poria cocos* (90 g) were added to the reaction mixture from the first step, followed by boiling for 1 h to prepare a cohesive agent. Finally, the cohesive agent was air-dried for 72 h and molded into pills. The pills were stored at 4°C, and the extract was dissolved in 1× PBS before conducting experiments. Ultra-high-performance liquid chromatography (UHPLC) was previously performed to determine each component, the content of principal markers, and the concentration of each component in the ALWPs formula, and the results of the quantitative analysis of four markers were presented in a previous study (Lee et al., 2018).

### Immunocytochemistry

Primary hippocampal neurons from E18 Sprague–Dawley rats were cultured as described previously (Brewer et al., 1993). To examine the effects of ALWPs on ADAMs, BACE1, or NEP levels, primary hippocampal neurons were transfected with GFP plasmid DNA for 24 h, followed by treatment with ALWPs (500 μg/ml) or PBS for 24 h. The primary hippocampal neurons were then fixed in ice-cold methanol for 8 min, washed with 1× PBS three times, and incubated with primary antibodies (α-ADAM 8, α-ADAM 9, α-ADAM 10, α-ADAM 12, α-ADAM 17, α-BACE1, or α-NEP) in GDB buffer (0.1% gelatin, 0.3% Triton X-100, 16 mM sodium phosphate pH 7.4, and 450 mM NaCl). The next day, the cells were washed three times with 1× PBS and incubated with secondary antibodies (Alexa Fluor 555 or Alexa Fluor 488) for 1 h at room temperature. Images were acquired using a confocal microscope (63×, Nikon, Japan), and ADAMs or BACE1 intensity was analyzed using ImageJ software. To measure the levels of ADAM17 and BACE1 in primary hippocampal neurons after ALWPs treatment, we used 18–19 neurons in four sister cultures per group to measure levels of ADAM17 (con, *n* = 67 dendrites; ALWPs, *n* = 71 dendrites) or BACE1 (con, *n* = 97 dendrites; ALWPs, *n* = 94 dendrites). To determine the effects of ALWPs on NEP levels in primary hippocampal neurons, we used 30 neurons in six sister cultures per group (con, *n* = 90 dendrites; ALWPs, *n* = 90 dendrites). To quantify the expression of ADAM17, BACE1, or NEP, the area of secondary dendritic segments was measured by drawing a region of interest (ROI) in a GFP fluorescence image using ImageJ software. Selected ROIs were overlaid on matching ADAM17, BACE1, or NEP images, and ADAM17, BACE1, or NEP fluorescence intensity inside the overlaid ROI was measured. ADAM17, BACE1, or NEP levels are presented as the measurement of the fluorescence intensity divided by the selected ROI area (Nwabuisi-Heath et al., 2012). To quantify the levels of ADAM8, ADAM9, ADAM10, or ADAM12 in primary hippocampal neurons after ALWPs treatment, we used 20 neurons in four sister cultures per group (ADAM8, AMAM9, ADAM10, ADAM12; con, *n* = 60 dendrites; ALWPs, *n* = 60 dendrites). Area of secondary dendritic segments were measured by drawing a ROI in red fluorescence image using ImageJ software. Fluorescence intensity inside the drawn ROI were measured, and ADAM8, ADAM9, ADAM10, or ADAM12 levels are presented as the measurement of the fluorescence intensity divided by the measured ROI area.

### Immunohistochemistry

ALWPs- or PBS-treated animals were perfused and fixed with 4% paraformaldehyde (PFA) solution, and brain tissues were flash-frozen and dissected using a cryostat (35 μm thickness). Sections were permeabilized for 1 h at room temperature in PBS with 0.2% Triton X-100 and 0.5% BSA, followed by incubation with primary antibodies (i.e., 4G8, AT100, AT180, Tau5) at 4 °C overnight. The sections were then washed three times with 0.5% BSA and incubated with secondary antibodies (Alexa Fluor 555 or Alexa Fluor 488, 1:500, Molecular Probes, Eugene, OR, USA) for 1 h at room temperature. The brain sections were mounted on gelatin-coated cover glass and covered with DAPI-containing mounting solution (Vector Laboratories, Burlingame, CA, USA). Images of the stained tissue sections were captured using a confocal microscope (TI-RCP, Nikon, Japan), and immunoreactivity was analyzed using ImageJ software. To measure the effects of ALWPs on cerebral amyloid plaque levels, we used six male 5x FAD mice per group, 12 in total. Three to four slices of each brain from −1.70 mm to −2.30 mm relative to the Bregma in stereotaxic coordinates were used to quantify amyloid plaques in the cortex and hippocampus. In addition, data were analyzed in a semi-automated manner using ImageJ software, and the results were confirmed by an independent researcher who did not participate in the current experiments. To determine whether ALWPs can modulate tau phosphorylation, 5x FAD mice (3 months old) were orally administered ALWPs (200 mg/kg, p.o.) or PBS daily for 2 weeks. After 2 weeks, we performed immunohistochemistry with anti-AT100, anti-AT180 or anti-Tau-5 antibodies. Images were acquired by a Nikon TI-RCP confocal laser microscope (10×). For this study, we used three male 5x FAD mice per group (six total), and three slices of each brain from −1.70 mm to −2.30 mm relative to the Bregma were used to quantify tau phosphorylation in the cortex and hippocampus. To quantify the levels of AT100, AT180, and Tau-5 in the cortex and hippocampus, the area of each region was measured by drawing a ROI in a DAPI fluorescence image using ImageJ software (*n* = 3 mice/group). The selected ROIs were overlaid on matching red fluorescence images, and the fluorescence intensity inside the overlaid ROIs was measured. The levels of AT100, AT180, and Tau-5 are presented as the measurement of the fluorescence intensity divided by the selected ROI area (Jensen, 2013).

### Cell-Surface Biotinylation and Live Cell-Surface Staining

APP-H4 cells (H4 cells overexpressing human APP and producing high levels of Aβ) were treated with ALWPs (500 μg/ml) or PBS for 24 h. Surface proteins were then labeled with sulfo-NHS-SS-biotin under gentle agitation at 4 °C for 30 min. Next, quenching solution (50 μl) was added, and the surface-labeled cells were lysed, disrupted by sonication, incubated for 30 min on ice, and clarified by centrifugation (10,000× *g*, 10 min). The supernatant was added to immobilized NeutrAvidin™ gel and incubated for 1 h at room temperature. The samples were washed with wash buffer (50 Mm Tris-HCl (pH 7.4), 0.15 M NaCl, 1% Nonidet P-40, and protease inhibitors) three times and incubated for 1 h in SDS-PAGE sample buffer including DTT at room temperature. Then, western blotting was performed with an antibody recognizing the N-terminus of APP. The effect of ALWPs on cell-surface levels of APP in primary hippocampal neurons was measured by live cell-surface staining as described previously (Hoe et al., 2006).

### APP Processing

APP-H4 cells (H4 cells overexpressing human APP and producing high levels of Aβ were treated with ALWPs (500 μg/ml) or PBS for 24 h, and conditioned medium and cell lysates were harvested. Secreted APP and APP C-terminal fragments (CTFs) were measured by western blotting with anti-sAPP alpha and anti-c1.6.1 antibodies (to detect full-length APP and APP CTF).

### Human Aβ ELISA

To examine the effects of ALWPs on human Aβ levels, we detected soluble human Aβ in conditioned medium with human Aβ 40 ELISA kits (KHB3481, Invitrogen, Carlsbad, CA, USA) according to the manufacturer’s instructions.

### Golgi Staining and Dendritic Spine Analysis

To analyze dendritic spine density in the brain, mice were orally administered PBS or ALWPs (200 mg/kg, p.o.) daily for 2 weeks. After 2 weeks, Golgi staining was performed using the FD Rapid GolgiStain Kit (FD NeuroTechnologies, Columbia, MD, USA) according to the protocol provided by the manufacturer. Briefly, PBS- or ALWPs-injected animals were submerged in solutions A and B for 2 weeks in the dark. After 2 weeks, brains were transferred to solution C in the dark for 24 h. Solution C was replaced after the first 24 h, and after an additional 24 h, individual mouse brains were sliced into coronal sections with a thickness of 150 μm using a VT1000S vibratome (Leica, USA). To quantify the dendritic spine density and morphologies in the cortical layer V and hippocampus CA1 regions, we used 8 slices of each mouse brain from −1.70 mm to −2.30 mm relative to the Bregma. Dendritic spines with a length of  0.4–3.8 μm and spine heads with a width of  0.3–2.9 μm were analyzed in this study.

### Statistical Analysis

All data were analyzed using either unpaired two-tailed *t*-tests with Welch’s correction for comparisons between two groups or one-way analysis of variance (ANOVA) for multiple comparisons with Prism 6 (GraphPad Software, San Diego, CA, USA). *Post hoc* analyses were completed with Tukey’s multiple comparison test, with significance set at *p* < 0.05. Data are presented as the mean ± standard error of the mean (SEM; *p < 0.05, **p < 0.01, ***p < 0.001).

## Results

### ALWPs Significantly Decrease Amyloid Plaque Levels in 5x FAD Mice

Several studies have shown that microglia are a possible cause of AD and other neurological disorders (Wee Yong, 2010; Hirsch et al., 2012; Asai et al., 2015; Heneka et al., 2015). In addition, we recently reported that ALWPs regulate LPS-induced neuroinflammation as well as LPS-induced cognitive function impairment (Lee et al., 2018). Based on the literature and our findings, we hypothesized that ALWPs may regulate AD pathology by modulating neuroinflammatory responses.

To test our hypothesis, we initially examined whether ALWPs can alter Aβ plaque levels. For this experiment, 5x FAD mice (3 months old) were orally administered ALWPs (200 mg/kg, p.o.) or PBS daily for 2 weeks. After 14 days, we performed immunohistochemistry with an anti-4G8 antibody (to detect amyloid plaques). Interestingly, we observed that oral administration of ALWPs in 5x FAD mice significantly reduced the amyloid plaque number in the cortex (Figures 1A,B) and hippocampus CA1 and DG (Figures 1C–E). These data suggest that ALWPs can modulate amyloid plaque loads in the cortex and hippocampus of 5x FAD mice.

Next, we tested whether ALWPs can alter Aβ levels. We transiently transfected COS7 cells (a monkey kidney fibroblast cell line) with a human APP expression construct for 24 h, followed by treatment with ALWPs (500 μg/ml) or PBS for 24 h, and Aβ ELISA was conducted. Surprisingly, ALWPs did not alter Aβ levels in COS7 cells compared to the control treatment (Supplementary Figures S1A,B). We then examined whether ALWPs can regulate Aβ levels in APP-H4 cells (H4 cells overexpressing human APP and producing high levels of Aβ APP-H4 cells were treated with ALWPs (500 μg/ml) or PBS for 24 h, and human Aβ ELISA was performed. We found that ALWPs significantly reduced human Aβ levels in APP-H4 cells compared to the control treatment (Figure 1F). These data suggest that ALWPs may differentially regulate human Aβ levels depending on the cell type or APP expression level (transiently overexpressed APP vs. stably overexpressed APP).

In addition, we examined whether ALWPs can alter APP processing to alter human Aβ levels. For this experiment, APP-H4 cells were treated with ALWPs (500 μg/ml) or PBS for 24 h, conditioned medium and cell lysates were collected, and western blotting was performed with anti-secreted APP alpha (sAPPα) and anti-c1.6.1 (to detect full-length APP and APP-CTF) antibodies. Interestingly, we observed that ALWPs-treated APP-H4 cells significantly increased sAPPα but did not alter the full-length APP and APP CTFs (Supplementary Figures S1C–F). These data suggest that ALWPs modulate α -secretase enzyme levels and lead to decreased Aβ levels/Aβ plaque load in APP-H4 cells.

### ALWPs Significantly Increase Cell-Surface Levels of APP

**Figure 2:**
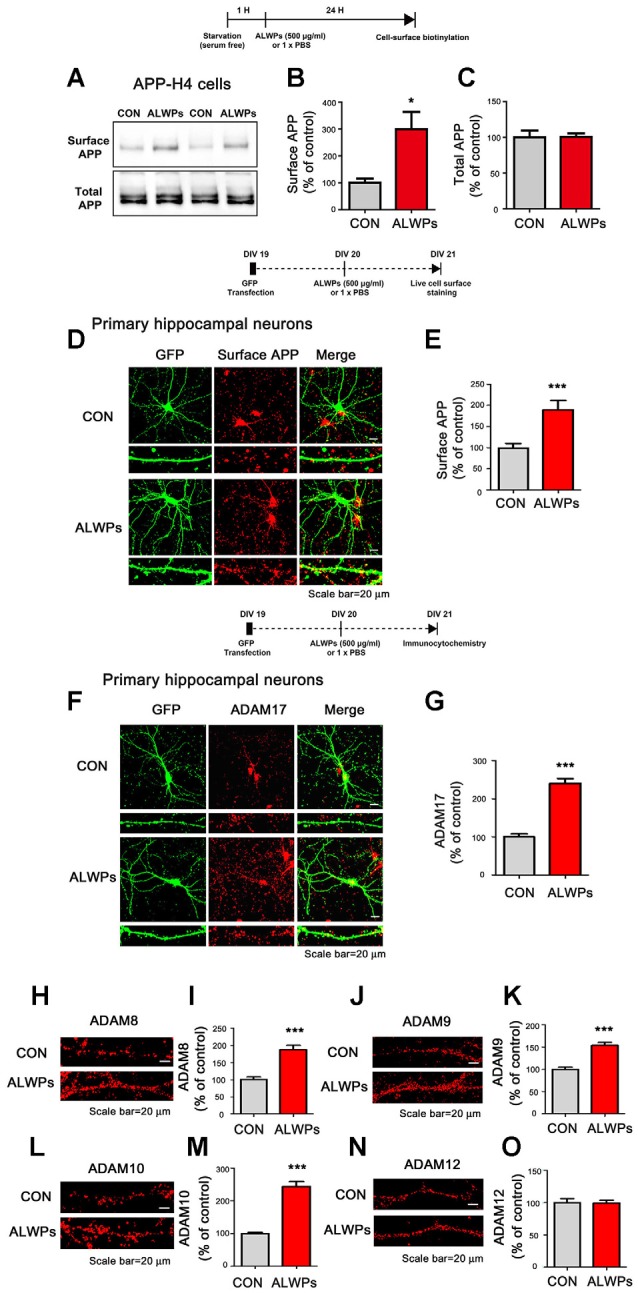
ALWPs significantly increase cell-surface levels of APP. **(A)** APP-H4 cells were treated with ALWPs (500 μg/ml) or PBS for 24 h, and cell-surface biotinylation assays were conducted with an anti-6E10 antibody. **(B,C)** Quantification of data from (**A**; surface APP and total APP; con, *n* = 8; ALWPs, *n* = 8). **(D,E)** Primary hippocampal neurons were transfected with GFP plasmid DNA for 24 h, followed by treatment with ALWPs (500 μg/ml) or PBS for 24 h and live cell-surface staining (con, *n* = 134 dendrites; ALWPs, *n* = 120 dendrites). **(F)** Primary hippocampal neurons were transfected with GFP plasmid DNA for 24 h, treated with ALWPs (500 μg/ml) or PBS for 24 h, and immunostained with an anti-ADAM17 antibody. **(G)** Quantification of data from (**F**; con, *n* = 67 dendrites; ALWPs, *n* = 71 dendrites). **(H,J,L,N)** Primary hippocampal neurons were treated with ALWPs (500 μg/ml) or PBS for 24 h and immunostained with anti-ADAM8, ADAM9, ADAM10, or ADAM12 antibodies. **(I,K,M,O)** Quantification of data from (**H,J,L,N**; con, *n* = 60 dendrites; ALWPs, *n* = 60 dendrites). *p < 0.05, ***p < 0.0001.

To determine the molecular mechanisms by which ALWPs regulate Aβ plaque load and Aβ levels, we first investigated the effects of ALWPs on cell-surface levels of APP, as several studies have shown that the α -secretases responsible for APP cleavage (i.e., ADAM17 and ADAM10) are mostly expressed on the cell surface (Cui et al., 2015). Thus, we hypothesized that ALWPs may affect cell-surface levels of APP, thereby promoting cleavage of APP by α -secretase and reducing Aβ levels. To test this hypothesis, APP-H4 cells were treated with ALWPs (500 μg/ml) or PBS for 24 h, and cell-surface levels of APP were measured using a cell-surface biotinylation assay. We found that ALWPs dramatically increased cell-surface levels of APP in APP-H4 cells (Figures 2A,B), but the total levels of APP were unchanged (Figures 2A,C).

To further confirm our findings, primary hippocampal neurons were transfected with GFP plasmid DNA (to visualize dendritic spines) for 24 h and treated with ALWPs (500 μg/ml) or PBS for 24 h. Then, live cell-surface immunostaining was conducted with an antibody targeting the N-terminus of APP. Again, ALWPs significantly increased cell-surface levels of APP in primary hippocampal neurons compared with the control treatment (Figures 2D,E). These data suggest that ALWPs may affect APP trafficking, resulting in decreased Aβ levels.

Since ALWPs decrease Aβ levels and increase cell-surface levels of APP, we further examined whether ALWPs alter levels of critical enzymes for APP processing. For these experiments, primary hippocampal neurons were transfected with GFP plasmid DNA for 24 h, treated with ALWPs (500 μg/ml) or PBS for 24 h, and immunostained with anti-ADAM17 (α-secretase) or anti-BACE1 (β-secretase) antibodies. We found that ALWPs significantly increased ADAM17 levels (Figures 2E,F) but not BACE1 levels (Supplementary Figures S2A,B).

We then examined whether ALWPs can alter the levels of other ADAMs with APP α-secretase cleavage activity (i.e., ADAM8, ADAM9, and ADAM10) as well as ADAM12, which is known to mediate Aβ neurotoxicity (Asai et al., 2003; Malinin et al., 2005; Vingtdeux and Marambaud, 2012). For these experiments, primary hippocampal neurons were treated with ALWPs (500 μg/ml) or PBS for 24 h and immunostained with anti-ADAM8, anti-ADMA9, anti-ADAM10, or anti-ADAM12 antibodies. We found that ALWPs significantly increased ADAM8, ADAM9, and ADAM10 levels compared to the control treatment (Figures 2H–M), but not ADAM12 levels (Figures 2N,O). These results indicate that ALWPs may modulate Aβ plaques by up-regulating α-secretase enzyme levels.

As another mechanism of action for decreasing Aβ plaques, we examined whether ALWPs can alter the level of the Aβ degradation enzyme neprilysin (NEP). For these experiments, primary hippocampal neurons were transfected with GFP plasmid DNA for 24 h, treated with ALWPs (500 μg/ml) or PBS for 24 h, and immunostained with an anti-NEP antibody. Interestingly, we observed that treatment with ALWPs increased NEP levels in primary hippocampal neurons (Supplementary Figures S2C,D).

Next, we tested whether ALWPs regulate the levels of Aβ degradation enzymes to alter Aβ plaque load and Aβ levels *in vivo*. 5x FAD mice (3 months old) were orally administered ALWPs (200 mg/kg, p.o.) or PBS daily for 2 weeks. After 14 days, we performed immunohistochemistry with an antibody against NEP. Interestingly, ALWPs increased NEP levels in the cortex (Supplementary Figures S2E,F) but not the hippocampus (Supplementary Figures S2G–I) compared with PBS-treated 5x FAD mice. These data suggest that ALWPs may modulate α -secretase enzyme levels and/or Aβ degradation enzyme levels in specific brain regions to alter Aβ pathology.

### Oral Administration of ALWPs Decreases Tau Phosphorylation in 5x FAD Mice

**Figure 3:**
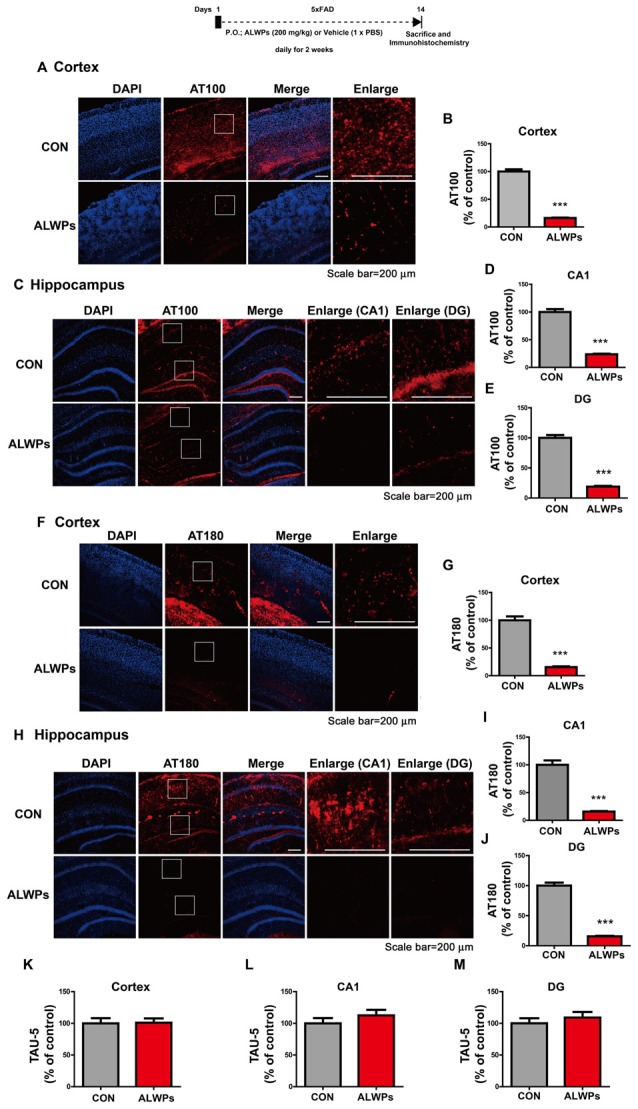
ALWPs significantly decrease tau phosphorylation in 5x FAD mice.** (A,C)** Representative images of the cortex **(A)** and hippocampus **(C)** of 5x FAD mice administered ALWPs (200 mg/kg, p.o.) or PBS immunohistochemically stained with an anti-AT100 antibody. **(B,D,E)** Quantification of data from (**A**; cortex; con, *n* = 3 mice; ALWPs, *n* = 3 mice) and (**C**; CA1 and DG; con, *n* = 3 mice; ALWPs, *n* = 3 mice). **(F–J)** Representative images of the cortex **(F)** and hippocampus **(H)** of 5x FAD mice immunohistochemically stained with an anti-AT180 antibody. **(G,I,J)** Quantification of data from (**F**; cortex; con, *n* = 3 mice; ALWPs, *n* = 3 mice) and (**H**; CA1 and DG; con, *n* = 3 mice; ALWPs, *n* = 3 mice). **(K–M)** Representative images of the cortex (**K**, con, *n* = 3 mice; ALWPs, *n* = 3 mice) and hippocampus CA1 (**L**, con, *n* = 3 mice; ALWPs, *n* = 3 mice) and DG (**M**, con, *n* = 3 mice; ALWPs, *n* = 3 mice) of 5x FAD mice immunohistochemically stained with an anti-Tau5 antibody. ***p < 0.0001.

As shown in Figures 1, 2, ALWPs reduce Aβ plaque load and Aβ levels by increasing cell-surface levels of APP. We, therefore, examined whether ALWPs can modulate tau phosphorylation, another hallmark of AD. 5x FAD mice (3 months old) were orally administered ALWPs (200 mg/kg, p.o.) or PBS daily for 2 weeks. After 14 days, immunohistochemistry was performed with anti-AT100, anti-AT180 or anti-Tau5 antibodies. Interestingly, we found that ALWPs significantly reduced tau phosphorylation at Thr212/Ser214 (AT100) in the cortex (Figures 3A,B) and hippocampus (Figures 3C–E). In addition, ALWPs significantly decreased immunoreactivity associated with tau phosphorylation at Thr231 (AT180) in the cortex (Figures 3F,G) and hippocampus (Figures 3H–J). However, ALWPs had no effect on total tau levels (anti-Tau5) compared to the control treatment (Figures 3K–M) in 5x FAD mice. These data suggest that ALWPs affect tau phosphorylation in a mouse model of AD.

### ALWPs Block the Impairment of Long-Term Memory by SCO in WT Mice

Several studies have demonstrated that enhanced pro-inflammatory cytokine levels and neuroinflammation can lead to synaptic dysfunction and memory impairment, which eventually result in neurodegenerative diseases (Fang et al., 2010; Tweedie et al., 2012). We recently reported that compared to the individual components, ALWPs have additive effects on LPS-induced neuroinflammation and LPS-mediated cognitive function impairment (Lee et al., 2018). Here, we further examined whether ALWPs can affect short-term and long-term memory in a SCO-induced amnesia animal model. SCO is a nonselective muscarinic receptor antagonist that induces learning and memory deficits similar to the aging-related and dementia-related symptoms of cognitive impairment (Lee et al., 2010).

**Figure 4:**
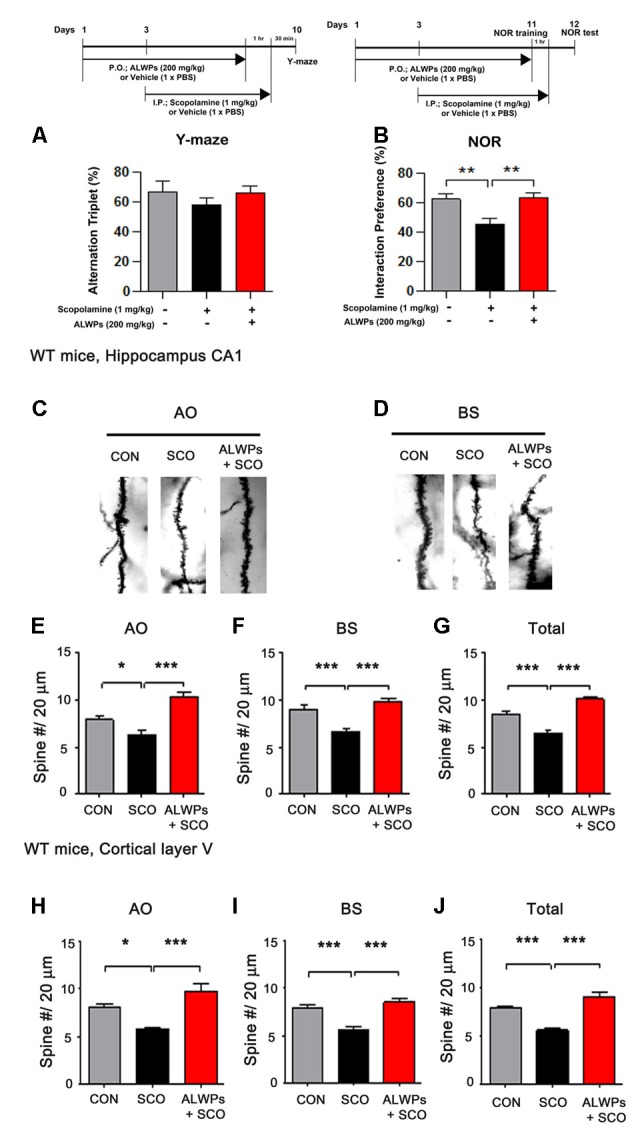
ALWPs reverse scopolamine (SCO)-induced long-term memory impairment in wild-type (WT) mice. **(A,B)** WT mice were orally administered ALWPs (200 mg/kg, p.o.) or PBS daily for 11 days. Beginning on day 3, the mice were injected with a control (PBS) or SCO (1 mg/kg) daily for 9 days, and Y-maze and novel object recognition (NOR) tests were performed on days 10 and 12, respectively (Y-maze: CON, *n* = 13; SCO, *n* = 13; SCO+ALWPs, *n* = 14; NOR: CON, *n* = 12; SCO, *n* = 12; SCO+ALWPs, *n* = 13). **(C,D)** Representative AO and BS dendrites from the hippocampal CA1 regions of mice treated with PBS (control) or ALWPs as indicated. **(E)** Dendritic spine density in hippocampal AO dendrites. **(F)** Dendritic spine density in hippocampal BS dendrites. **(G)** Total average dendritic spine density in hippocampal dendrites (*n* = 4 mice/group, con, *n* = 27–28/neurons ; SCO, *n* = 27–30/neurons ; ALWPs + SCO, *n* = 27–28/neurons). **(H)** Dendritic spine density in cortical layer V AO dendrites. **(I)** Dendritic spine density in cortical layer V BS dendrites. **(J)** Total average dendritic spine density in cortical layer V dendrites (*n* = 4 mice/group, con, *n* = 27–29/neurons ; SCO, *n* = 32–33/neurons ; ALWPs + SCO, *n* = 29–30/neurons). *p < 0.05, **p < 0.01, ***p < 0.0001.

To investigate whether ALWPs can attenuate the learning and memory impairment induced by SCO, WT mice were orally administered a control (PBS) or ALWPs (200 mg/kg, p.o.) daily for 11 days. Beginning on day 3, SCO [1 mg/kg, intraperitoneal injection (i.p.)] was injected daily for 9 days (days 3–11), and Y-maze and NOR tests were conducted on days 10 and 12, respectively. In the Y maze, spontaneous “alternation triplet” behavior, defined as successive visits of the three arms in order (e.g., A-B-C or B-A-C or C-A-B), was quantified to examine short-term memory. There was no difference in alternation behavior in the Y-maze between the groups (Figure 4A). For the NOR test, the “interaction preference” for the novel object, which is the relative interaction time with the novel object compared to the total interaction time with both the novel and familiar objects, was determined 24 h after the NOR training session to examine the level of long-term object recognition memory. We found that WT mice injected with SCO exhibited significantly decreased long-term memory compared with control WT mice (Figure 4B). Interestingly, treatment of WT mice with ALWPs prior to SCO injection blocked the impairment of long-term memory compared with SCO-injected WT mice (Figure 4B).

We then tested whether ALWPs can alter dendritic spine formation, which is associated with learning and memory. WT mice were orally administered a control (PBS) or ALWPs (200 mg/kg, p.o.) daily for 11 days. Beginning on day 3, SCO (1 mg/kg, i.p.) was injected daily for 9 days (days 3–11), and Golgi staining was performed. Interestingly, we observed that the dendritic spine density was significantly reduced in SCO-injected WT mice compared to control WT mice in hippocampus CA1 (Figures 4C–G). However, pretreatment of WT mice with ALWPs before SCO treatment significantly rescued the dendritic spine number in hippocampus CA1 compared to SCO-injected WT mice (Figures 4C–G). In addition, pretreatment with ALWPs also significantly recovered the dendritic spine number in the cortical layer V region compared to SCO-injected WT mice (Figures 4H–J). These results indicate that ALWPs can regulate cognitive performance by altering dendritic spine density in SCO-injected WT mice.

### ALWPs Promote Long-Term Memory and Dendritic Spine Density in 5x FAD Mice

**Figure 5:**
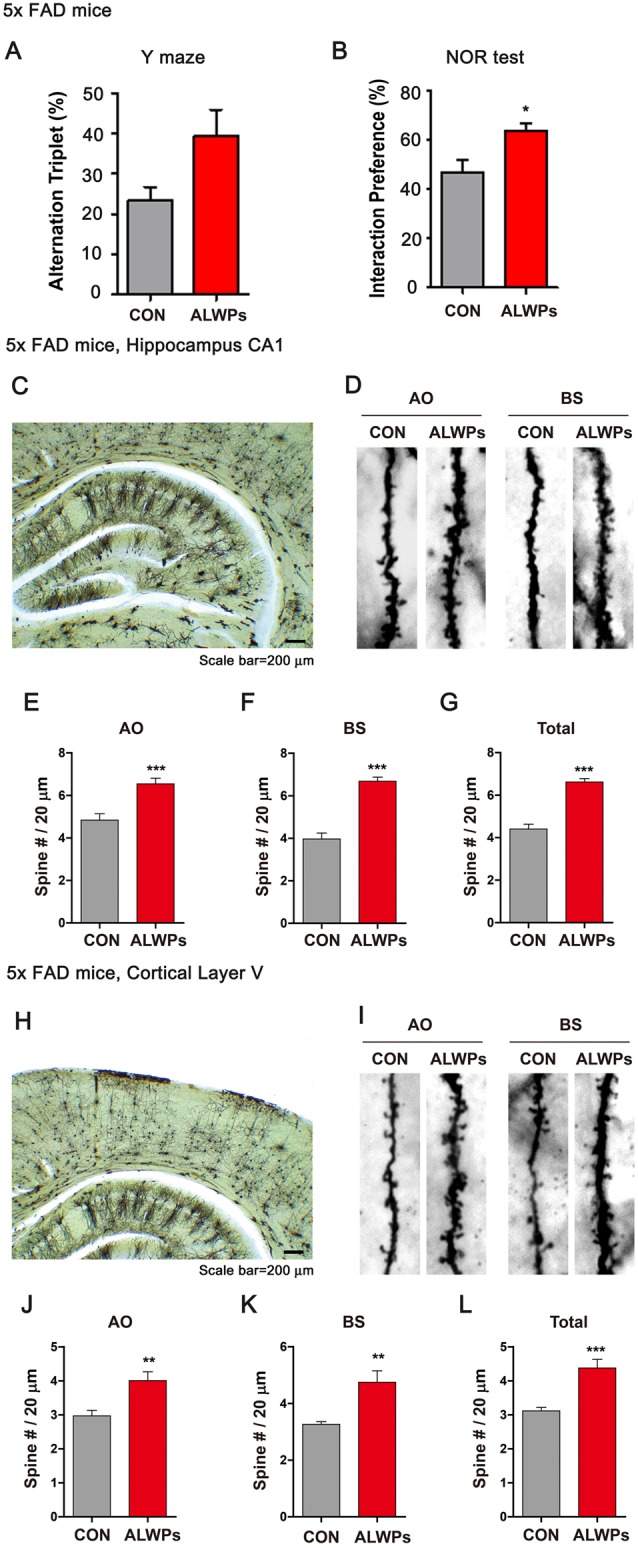
Oral administration of ALWPs to 5x FAD mice significantly enhances long-term memory and dendritic spine number.** (A,B)** 5x FAD mice were orally administered ALWPs (200 mg/kg, p.o.) or PBS (control) daily for 2 weeks. After 12 days, Y-maze (5x FAD CON, *n* = 8 mice; 5x FAD ALWPs, *n* = 8 mice) and NOR (5x FAD CON, *n* = 8 mice; 5x FAD ALWPs, *n* = 8 mice) tests were performed. **(C,D)** Representative AO and BS dendrites from hippocampal CA1 neurons of 5x FAD mice treated with PBS or ALWPs (200 mg/kg, p.o.) as indicated. **(E–G)** Dendritic spine density in hippocampal AO dendrites **(E)** and BS dendrites **(F)** and total average spine density **(G)** in hippocampal dendrites of 5x FAD mice (*n* = 8 mice/group, CA1: con, *n* = 58–72/neurons; ALWPs, *n* = 68–83/neurons). **(H,I)** Representative AO and BS dendrites from cortical layer V neurons of 5x FAD mice treated with PBS or ALWPs (200 mg/kg, p.o.) as indicated. **(J–L)** Dendritic spine density in cortical layer V AO dendrites **(J)** and BS dendrites **(K)** and total average spine density **(L)** in cortical dendrites of 5x FAD mice (*n* = 8 mice/group, con, *n* = 36–42/neurons ; ALWPs, *n* = 36–37/neurons). *p < 0.05, **p < 0.01, ***p < 0.0001.

As shown in Figure 4, ALWPs can regulate cognitive function and dendritic spine formation. We next examined whether ALWPs can affect cognitive performance in a mouse model of AD. For this experiment, 5x FAD mice (3 months old) were administered vehicle (PBS) or ALWPs (200 mg/kg, p.o.) orally daily for 2 weeks, and Y-maze and NOR tests were conducted. Interestingly, 5x FAD mice treated with ALWPs exhibited increased short-term memory and significantly improved long-term memory compared with PBS-administered 5x FAD mice (Figures 5A,B). These data suggest that ALWPs can also modulate cognitive function in a mouse model of AD.

Next, we investigated whether ALWPs can regulate dendritic spine number in a mouse model of AD. For this experiment, 3-month-old 5x FAD mice were administered ALWPs (200 mg/kg, p.o.) or PBS orally daily for 2 weeks. After 14 days, we conducted Golgi staining and measured dendritic spine number and spine morphology. We found that ALWPs significantly increased dendritic spine density in the hippocampus (Figures 5C–G) and in cortex layer V (Figures 5H–L). We then examined whether dendritic spine morphology was altered in ALWPs-treated 5x FAD mice and found shorter and narrow spines in apical oblique (AO) dendrites of hippocampus CA1 compared to the control group (Supplementary Figure S3). However, treatment with ALWPs did not alter dendritic spine morphology in basal (BS) dendrites of hippocampus CA1 or cortical layer V (Supplementary Figure S3), suggesting that ALWPs selectively affect dendritic spine morphology and density in specific brain regions in a mouse model of AD.

### ALWPs Significantly Increase Dendritic Spine Number in Primary Hippocampal Neurons and Wild-Type Mice

**Figure 6:**
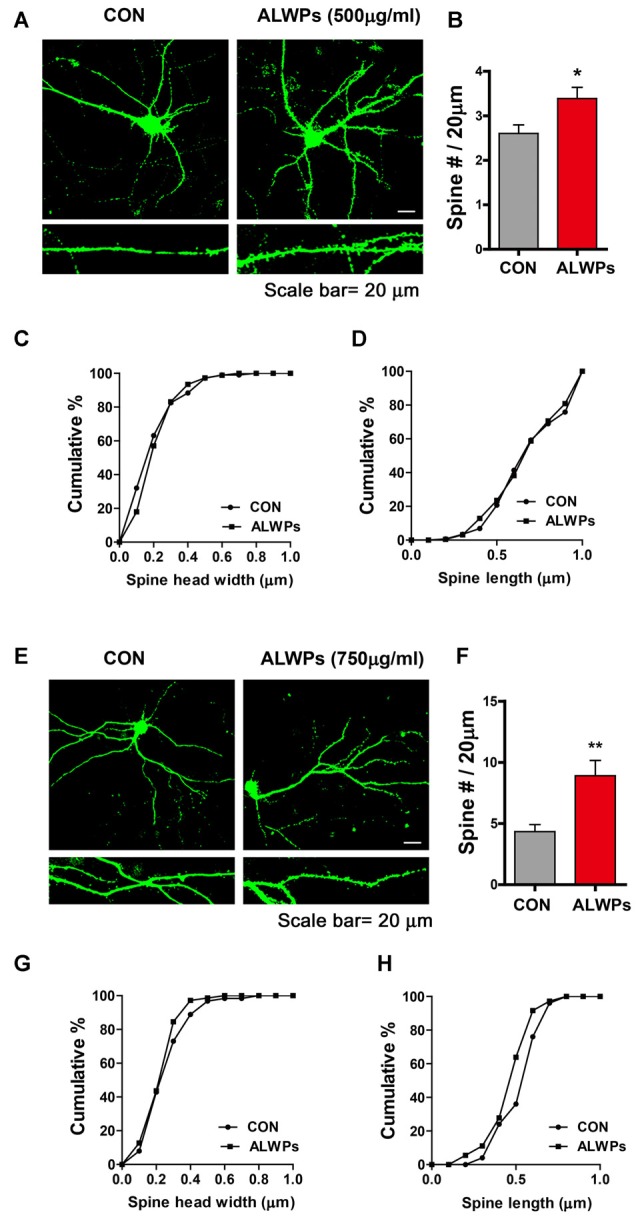
ALWPs significantly promote spinogenesis in primary hippocampal neurons. **(A)** Primary hippocampal neurons were transfected with GFP plasmid DNA, followed by treatment with ALWPs (500 μg/ml) or PBS for 24 h and measurement of dendritic spine density. **(B)** Quantification of data from (**A**; CON, *n* = 29; ALWPS, *n* = 27). **(C,D)** The cumulative distribution percentage of spine head width and spine length in primary hippocampal neurons treated with ALWPs (500 μg/ml) or PBS for 24 h (Kolmogorov–Smirnov test). **(E)** Primary hippocampal neurons were transfected with GFP plasmid DNA, followed by treatment with ALWPs (750 μg/ml) or PBS for 24 h and measurement of dendritic spine density. **(F)** Quantification of data from (**F**; CON, *n* = 34; ALWPS, *n* = 26). **(G,H)** The cumulative distribution percentage of spine head width and spine length in primary hippocampal neurons treated with ALWPs (750 μg/ml) or PBS for 24 h (Kolmogorov–Smirnov test). *p < 0.05, **p < 0.01.

As ALWPs affect cognitive function as well as dendritic spine formation in SCO-injected WT mice and 5x FAD mice, we next examined whether ALWPs can alter dendritic spine formation under normal conditions. For this experiment, primary hippocampal neurons (DIV19) were transfected with GFP plasmid DNA (to visualize dendrite segments and spines) and treated with ALWPs (500 μg/ml) or control (PBS) for 24 h. Dendritic spine density was significantly increased in 500 μg/ml ALWPs-treated primary hippocampal neurons (Figures 6A,B). However, ALWPs (500 μg/ml) did not alter spine morphology (i.e., dendritic spine head width and spine length) in primary hippocampal cultures (Figures 6C,D).

We then tested whether a higher concentration of ALWPs can alter dendritic spine number. Primary hippocampal neurons (DIV19) were transfected with GFP plasmid DNA and treated with ALWPs (750 μg/ml) or control (PBS) for 24 h. After 24 h, dendritic spine density was measured. Dendritic spine density was significantly increased in primary hippocampal neurons treated with 750 μg/ml ALWPs (Figures 6E,F), but spine head width and spine length were not altered (Figures 6G,H). These data suggest that ALWPs can increase the number of dendritic spines in primary hippocampal neurons in mature stages.

**Figure 7:**
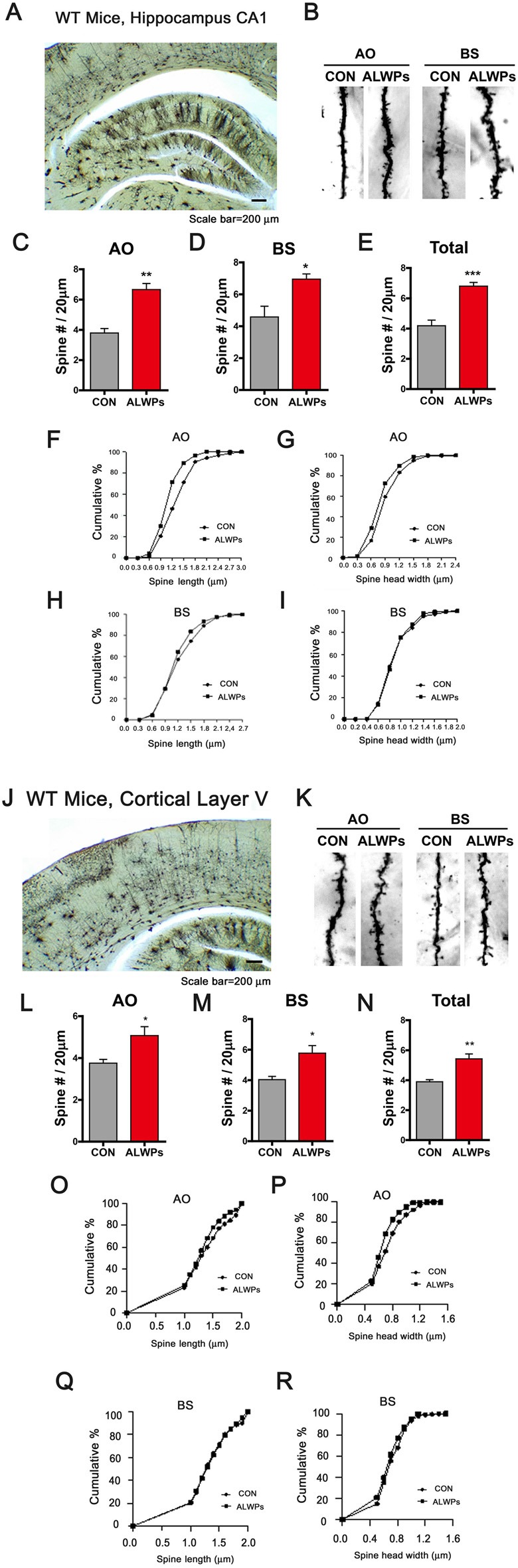
Oral administration of ALWPs to WT mice significantly promotes dendritic spine formation. **(A,B)** Representative AO and BS dendrites from hippocampal CA1 neurons of WT mice treated with the control or ALWPs (200 mg/kg, p.o.) as indicated. **(C–E)** Dendritic spine density in hippocampal AO dendrites **(C)** and BS dendrites **(D)** and total average spine density **(E)** in hippocampal dendrites of WT mice (*n* = 4 mice/group, con, *n* = 20–37/neurons ; ALWPs, *n* = 27–36/neurons). **(F,G)** The cumulative distribution percentage of spine length **(F)** and spine head width **(G)** in hippocampal CA1 AO dendrites of WT mice (Kolmogorov–Smirnov test). **(H,I)** The cumulative distribution percentage of spine length **(H)** and spine head width **(I)** in hippocampal CA1 BS dendrites of WT mice (Kolmogorov–Smirnov test). **(J–K)** Representative AO and BS dendrites from cortical layer V neurons of WT mice treated with the control or ALWPs (200 mg/kg, p.o.), as indicated. **(L–N)** Dendritic spine density in cortical layer V AO dendrites **(L)** and BS dendrites **(M)** and total average spine density **(N)** in cortical dendrites of WT mice (*n* = 4 mice/group, con, *n* = 42–44/neurons ; ALWPs, *n* = 42–43/neurons). **(O,P)** The cumulative distribution percentage of spine length **(O)** and spine head width **(P)** in cortical layer V AO dendrites of WT mice (Kolmogorov–Smirnov test). **(Q–R)** The cumulative distribution percentage of spine length **(Q)** and spine head width **(R)** in cortical layer V BS dendrites of WT mice (Kolmogorov–Smirnov test). *p < 0.05, **p < 0.01, ***p < 0.0001.

To further examine whether ALWPs can alter dendritic spine density in WT mice, WT mice (3 months old) were orally administered ALWPs (200 mg/kg, p.o.) or PBS daily for 2 weeks. After 14 days, we conducted Golgi staining and measured dendritic spine number and spine morphology. Treatment with ALWPs significantly increased the dendritic spine number in AO and BS dendrites in the hippocampus (Figures 7A–E). In addition, ALWPs-treated WT mice had longer and wider spines in hippocampal AO dendrites but not hippocampal BS dendrites compared with control WT mice (Figures 7F–I).

We then tested whether ALWPs can affect dendritic spine density and morphology in cortex layer V. The dendritic spine density in cortical layer V was significantly increased in ALWPs-treated WT mice (Figures 7J–N). Moreover, the spines in cortical layer V AO dendrites were longer and wider in ALWPs-treated WT mice compared with control WT mice (Figures 7O,P). However, neither the length nor width of dendritic spines changed in cortical layer V BS dendrites (Figures 7Q,R). Taken together, these data suggest that ALWPs can also affect dendritic spine number and spine morphology in WT mice.

### ALWPs Promote Dendritic Spine Density in an ERK-Dependent Manner

**Figure 8:**
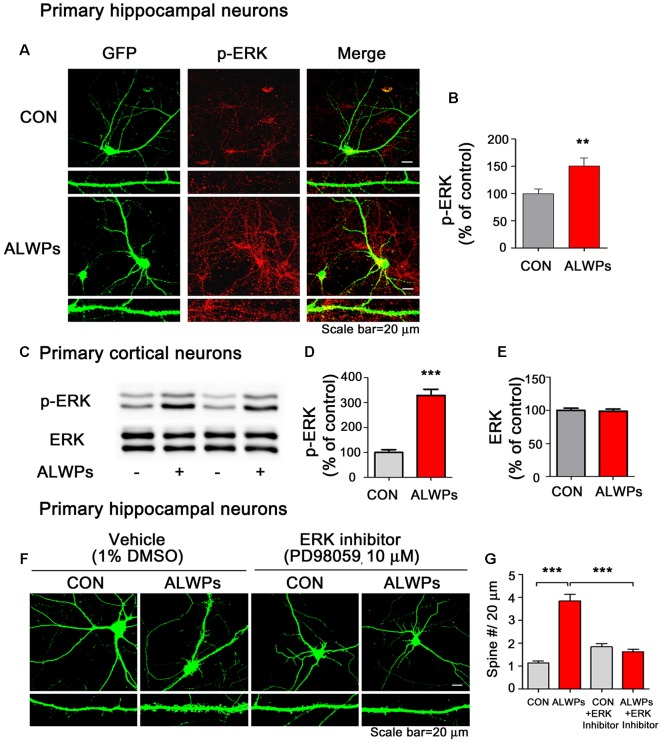
ALWPs require extracellular signal-regulated kinase (ERK) signaling to modulate dendritic spine formation. **(A)** Primary hippocampal neurons were transfected with GFP plasmid DNA for 24 h, treated with ALWPs (500 μg/ml) or PBS for 24 h, and immunostained with an anti-p-ERK antibody. **(B)** Quantification of data from (**A**; con, *n* = 88 dendrites; ALWPs, *n* = 100 dendrites). **(C)** Primary cortical neurons were treated with ALWPs (500 μg/ml) or PBS for 24 h and immunoblotted with anti-p-ERK or anti-ERK antibodies. **(D,E)** Quantification of data from (**C**; p-ERK, *n* = 8; ERK, *n* = 4). **(F)** Primary hippocampal neurons were transfected with GFP plasmid DNA for 24 h, pretreated with an ERK inhibitor (PD98059, 10 μM) or vehicle (1% DMSO) for 1 h, and treated with ALWPs (500 μg/ml) or PBS for 24 h, followed by measurement of dendritic spine density. **(G)** Quantification of data from (**F**; con, *n* = 86; ALWPs, *n* = 71; PD98059, *n* = 84; ALWPS+PD98059, *n* = 91). **p < 0.01, ***p < 0.0001.

Ras/ERK signaling is involved in dendritic spine formation (Thomas and Huganir, 2004; Tang and Yasuda, 2017). Therefore, we tested whether ALWPs affect ERK signaling. To address this question, we transfected primary hippocampal neurons with GFP plasmid DNA, treated the neurons with ALWPs (500 μg/ml) or control (PBS) for 24 h, and immunostained the neurons with an anti-p-ERK antibody. p-ERK levels were significantly increased in ALWPs-treated primary hippocampal neurons compared to the control treatment (Figures 8A,B). As a complementary study, primary cortical neurons were treated with ALWPs (500 μg/ml) or control (PBS) for 24 h, and western blotting was performed with anti-p-ERK and anti-ERK antibodies. Again, we observed that treatment with ALWPs significantly increased p-ERK levels in primary cortical neurons (Figures 8C,D), whereas the total levels of ERK were unchanged (Figures 8C,E).

Next, we examined whether ALWPs require ERK signaling to alter dendritic spine density. For this experiment, primary hippocampal neurons were transfected with GFP plasmid DNA for 24 h, pretreated with an ERK inhibitor (PD98059, 10 μM) for 1 h, and treated with ALWPs (500 μg/ml) or control (PBS) for 24 h. After 24 h, the dendritic spine number was measured. Treatment with ALWPs significantly increased the dendritic spine number, consistent with our previous findings (Figures 8F,G). By contrast, ALWPs did not increase the dendritic spine number in neurons pretreated with the ERK inhibitor (Figures 8F,G). These data suggest that ERK signaling is necessary for ALWPs to regulate dendritic spine formation.

We then investigated whether ALWPs can affect Rap signaling, which is involved in dendritic spine retardation, to modulate dendritic spine formation (Lee K. J. et al., 2011; Morel et al., 2018). Primary cortical neurons were treated with ALWPs (500 μg/ml) or control (PBS) for 24 h, and western blotting was performed with anti-PLK2, anti-RapGEF, and anti-p-JNK/JNK antibodies. We found that ALWPs did not alter any Rap signaling pathway in primary cortical neurons (Supplementary Figure S4).

## Discussion

In traditional oriental herbal medicine, LWPs have been used for more than a 1,000 years to improve or restore declining cognitive function related to the aging process and geriatric diseases (Wee Yong, 2010). LWPs have been shown to modulate the neuronal and synaptic function and to enhance cognitive function by improving functional neurotransmission between neurons (Dong et al., 2018; Lee et al., 2018). Another traditional herbal medicine, antler, is one of the most effective drugs in ameliorating the effects of aging and fatigue and is believed to have many health benefits (Wu et al., 2013). Based on the literature, we recently developed ALWPs by combining LWPs with antler and reported that ALWPs can regulate the LPS-induced neuroinflammatory response and rescue LPS-induced short-and long-term memory impairment (Lee et al., 2018). In the present study, we further examined whether ALWPs can affect AD pathology and cognitive function under normal and pathological conditions. Interestingly, we observed that ALWPs altered both Aβ plaque load and tau phosphorylation in a mouse model of AD. In addition, ALWPs regulated cognitive function by modulating dendritic spine number in SCO-injected WT mice and in 5x FAD mice. Moreover, ALWPs affected dendritic spine formation through ERK signaling in primary hippocampal neurons. Taken together, our results suggest that ALWPs may hold therapeutic drug potential for AD by modulating AD pathology and cognitive function impairment.

Several studies have reported that the individual components of LWPs have effects on Aβ pathology (Sangha et al., 2012). For example, *Corni fructus* has inhibitory activity against BACE1 (β-secretase) and decreases Aβ levels (Youn and Jun, 2012). In another study, Yangxue Qingnao (YXQN), which shares some of the components of ALWPs, significantly decreased cerebral Aβ plaque loads by attenuating the activity of BACE1 (Wang et al., 2017). However, whether the individual components of LWPs or ALWPs are able to affect AD pathology and the associated molecular mechanisms have not been explored in detail. Here, we demonstrated that ALWPs treatment significantly reduced amyloid plaque loads in 5x FAD mice (Figure 1) and Aβ levels in APP-H4 cells (Figure 1).

How do ALWPs regulate Aβ pathology? Based on our findings and the literature, one possible mechanism is that ALWPs increase cell-surface levels of APP, leading to increased cleavage of α-secretase on the cell surface and thereby regulating Aβ levels. Indeed, we found that ALWPs significantly enhanced cell-surface levels of APP in APP-H4 cells and primary hippocampal neurons (Figure 2). In addition, ALWPs increased ADAM8, ADAM9, ADAM10, and ADAM17 (α -secretase) but not BACE1 (β-secretase) and ADAM12 levels in primary hippocampal neurons (Figure 2, Supplementary Figure S2), suggesting that ALWPs regulate Aβ pathology by modulating APP trafficking and/or Aβ pathology-related enzymes/proteins. Another possible mechanism by which ALWPs regulate Aβ pathology is by regulating the levels/activity of Aβ degradation enzymes, specifically NEP, to reduce Aβ pathology. Several studies have shown that NEP, which is also known as neutral endopeptidase, play important roles in AD pathogenesis (Iwata et al., 2001; Turner et al., 2001). For instance, NEP expression levels decline with aging, leading to decreased Aβ clearance (Grimm et al., 2013), and NEP mRNA and protein levels are significantly lower in AD brains than in age-matched normal control brains (Wang et al., 2010). This decline in NEP levels is considered an important factor in the progression of AD. In the present study, we observed that ALWPs treatment increased NEP levels in primary hippocampal neurons. In addition, we found that oral administration of ALWPs to 5x FAD mice significantly increased NEP levels in the cortex but not the hippocampus (Supplementary Figure S2). The duration of treatment of 5x FAD mice with ALWPs may not have been sufficient to greatly affect NEP levels in the brain; thus, future studies will use longer treatment times to examine the effects of ALWPs on NEP levels in the brain. Overall, our data suggest that ALWPs alter Aβ pathology by regulating APP trafficking and/or APP processing enzyme levels/activity.

Since, we observed that ALWPs can regulate Aβ pathology, we examined the effects of ALWPs on tau phosphorylation. Tau has many sites of phosphorylation with distinct functions. For example, tau phosphorylation at Thr212, Thr231, and Ser262 produces a toxic tau molecule, induces caspase activation, and leads to neurodegeneration (Alonso et al., 2010). Tau phosphorylation at Thr212/Ser214 (AT100) may facilitate microtubule-based trafficking of organelles by causing detachment of tau from microtubules (Stamer et al., 2002; Ksiezak-Reding et al., 2003). In addition, neurofibrillary tangle (NFT) formation is tightly correlated with the phosphorylation of tau at Thr212/Ser214 (AT100) and Thr 231 (AT180) and can induce many neurodegenerative diseases (Augustinack et al., 2002; Congdon and Sigurdsson, 2018). Although the precise pathophysiology is not fully known, evidence indicates that tau phosphorylation at Thr212/Ser214 and Thr231 may be an important therapeutic target for AD. Interestingly, we found that ALWPs significantly decreased tau phosphorylation at Thr212/Ser214 (AT100) and Thr231 (AT180) in 5x FAD mice (Figure 3).

How do ALWPs regulate both Aβ pathology and tau phosphorylation? Several recent studies suggest that Aβ and tau can act synergistically in AD. For instance, Aβ promotes tau phosphorylation at specific tau epitopes as well as neurodegeneration (Jin et al., 2011). Consistent with these findings, another recent study demonstrated that Aβ upregulates tau hyperphosphorylation as well as tau kinases, including GSK-3β, cyclin-dependent kinase 5 (CDK-5), proline-directed kinase (PDK), and casein kinase II, *in vitro* and in a model of AD (Takashima et al., 1993; Vintém et al., 2009; Oliveira et al., 2015; Wu et al., 2018). Conversely, other studies have shown that tau can affect Aβ pathology. For example, Leroy et al. (2012) showed that APP/PS1 mice in which the tau protein was deleted exhibited a significantly reduced Aβ plaque number compared with APP/PS1/Tau transgenic mice. Supporting these findings, Rapoport et al. (2002) found that Aβ did not induce neurite degeneration in primary neurons lacking tau, whereas treatment with human tau protein rescued the Aβ sensitivity of primary neurons lacking tau. However, the mechanisms by which Aβ and/or tau modulates tau phosphorylation and/or Aβ pathology have not been comprehensively studied. In the present study, we observed that ALWPs decreased both Aβ plaque burden and tau phosphorylation in 5x FAD mice. Based on the literature and our findings, there are several possible routes by which ALWPs might regulate both Aβ and tau phosphorylation. One possibility is that ALWPs first affect Aβ pathology, which in turn leads to regulation of tau kinase activity (i.e., CDK5, GSK-3B) and modulation of tau phosphorylation. A second possibility is that ALWPs may alter tau kinase activity as well as tau phosphorylation, with subsequent modulating effects on Aβ pathology. It is also possible that ALWPs regulate Aβ or tau pathology *via* independent mechanisms by modulating tau- or Aβ-related downstream targets. Thus, future studies will explore how ALWPs influence the relationship between Aβ and tau pathology as well as the molecular mechanisms by which ALWPs regulate Aβ and tau pathology.

The processes of learning and memory formation depend on synaptic plasticity, which appears to mediate a behavioral change caused by an experience and the process for acquiring memory. Dendritic spines in the hippocampal and cortical regions are considered the major factor in synaptic plasticity for memory formation (Leuner et al., 2003; Restivo et al., 2009). The relationship between dendritic spine density and memory behaviors has been demonstrated using various disease models and different behavioral assessments (Chen et al., 2010; Jiang et al., 2015; Roy et al., 2016, 2017). The major symptoms observed in aging and AD patients include memory decline or synapse and dendritic spine loss, possibly due to progressive impairments of synaptic circuits or plasticity (Knobloch and Mansuy, 2008; Dorostkar et al., 2015). The potential use of traditional herbal materials to improve memory function related to aging and neurodegenerative diseases has received extensive attention (Shih et al., 2000; Li et al., 2016; Kuboyama et al., 2017). For instance, LWPs and their analogs (i.e., LDW and LDW-AFC) modulate the synaptic function and ameliorate impaired learning and memory performance in animal models of cognitive deficit such as AD. Other studies have demonstrated that LWPs facilitate the induction of LTP in SAMP8 hippocampal slices by inhibiting voltage-dependent calcium channels (VDCCs; Huang et al., 2012). In addition, *Rehmanniae glutinosa*, a component of LWPs, attenuates SCO-induced dementia and accompanying learning and memory impairment *via* regulation of cholinergic systems in rat (Lee B. et al., 2011). *Rehmanniae radix* (Qifu-yin), a component of LWPs, has also been shown to affect cognitive function in mice (Wang et al., 2014). Another traditional herbal medicine, PLAG (a component of antler), ameliorates SCO-induced memory impairment in mice *via* acetylcholinesterase inhibition and LTP activation (Jeon et al., 2017). However, whether LWPs or other traditional herbal medicines can regulate dendritic spine formation, which is associated with learning and memory, has not been well-studied. In the current study, we examined whether ALWPs can affect learning and memory as well as dendritic spine formation. We observed that ALWPs attenuated the impairment of long-term memory in SCO-injected WT mice (Figure 4) and in 5x FAD mice (Figure 5). In addition, ALWPs rescued dendritic spine density in SCO-injected WT mice (Figure 4) and in 5x FAD mice (Figure 5), promoted dendritic spine formation in cultured hippocampal neurons (Figure 6), and increased the dendritic spine number in WT mice (Figure 7). Interestingly, we observed altered dendritic spine morphology in hippocampal AO dendrites but not hippocampal BS dendrites in ALWPs-treated 5x FAD or WT mice (Supplementary Figure S3, Figure 6). These phenotypes suggest that several pathways may be regulated by ALWPs in hippocampal AO dendrites. Further study of the differences in dendritic spine morphology between hippocampus CA1 AO and BS dendrites in mice orally administered ALWPs is needed.

To elucidate the molecular mechanism by which ALWPs regulate the formation of dendritic spines to affect learning and memory processes, we first tested whether ALWPs can modulate the Ras and Rap signaling pathways. Ras and Rap signaling are closely related to dendritic spine formation/retardation (Lee K. J. et al., 2011; Tang and Yasuda, 2017), and several studies have shown that Ras activity is involved in Aβ pathology as well as synaptic function (Passafaro et al., 2003; Qin et al., 2005; Gu and Stornetta, 2007; Szatmari et al., 2013). Therefore, it is important to develop therapeutic agents that improve learning and memory by modulating both the Ras and Rap signaling pathways. Here, we found that ALWPs significantly upregulated levels of ERK, an important molecule in the Ras signaling pathway (Figure 8). In addition, treatment with an ERK inhibitor blocked the effects of ALWPs on dendritic spine formation, suggesting that ALWPs may regulate dendritic spine formation *via* ERK signaling. We also tested whether ALWPs alter the Rap signaling pathway to regulate dendritic spine formation as another possible mechanism. We found that ALWPs did not alter any Rap signaling molecule, including Plk2, RapGEF, and JNK (Supplementary Figure S4). Overall, our data suggest that ALWPs regulate dendritic spine formation through the ERK signaling pathway rather than the Rap signaling pathway.

## Conclusion

In summary, our findings showed that ALWPs regulated Aβ plaque load and tau phosphorylation in a mouse model of AD. In addition, ALWPs rescued the impairment of long-term memory in SCO-injected WT mice and 5x FAD mice. Moreover, ALWPs promoted dendritic spine formation in cultured hippocampal neurons and significantly increased dendritic spine density in WT mice, SCO-injected mice, and 5x FAD mice. Finally, ALWPs altered dendritic spine formation through an ERK-dependent mechanism. We, therefore, advocate ALWPs as a useful therapeutic agent for the treatment of AD.

## Ethics Statement

All animal experiments were performed in accordance with approved animal protocols and guidelines established by KBRI (IACUC-2016-0013).

## Author Contributions

YK, JWK, Y-MW, and H-SH: study conception and design. YN, BJ, YK, and JK: acquisition of data. YN, BJ, J-YL, K-MH, and K-YR: preparation of figures. YN, BJ, YK, JK, JWK, Y-MW, and H-SH: writing of manuscript.

## Conflict of Interest Statement

The authors declare that the research was conducted in the absence of any commercial or financial relationships that could be construed as a potential conflict of interest.
