# Supplementary material for: ALWPs Improve Cognitive Function and Regulate Aβ Plaque and Tau Hyperphosphorylation in a Mouse Model of Alzheimer’s Disease
Source: Front Mol Neurosci. 2019 Aug 16;12:192. doi: 10.3389/fnmol.2019.00192 (PMC6707392; doi:10.3389/fnmol.2019.00192)
Supplement: Supplementary file 1 [file Data_Sheet_1.docx]

**Supplementary Figures**

**ALWPs improve cognitive function and regulate Aβ plaque and tau hyperphosphorylation in a mouse model of Alzheimer’s disease**

Youngpyo Nam^1,5^, Bitna Joo^1,4,5^, Ju-Young Lee^1^, Kyung-Min Han^1,4^, Ka-Young Ryu^1^, Young Ho Koh^2^, Jeongyeon Kim^1,^, Ja Wook Koo^1,4^, Young-Man We^3,*^, Hyang-Sook Hoe^1,4,*^

^1^Department of Neural Development and Disease, Korea Brain Research Institute (KBRI), 61 Cheomdan-ro, Dong-gu, Daegu, Korea, 41068; ^2^Division of Brain Disease, Center for Biomedical Sciences, Center for Infectious Diseases, Korea National Institute of Health, Osong-eup, Heungdeok-gu, Republic of Korea; ^3^College of Korean Medicine, Wonkwang University, Iksandae-ro, Iksan, Jeonbuk, Korea, 54538; ^4^Department of Brain & Cognitive Sciences, Daegu Gyeongbuk Institute of Science & Technology (DGIST), 333 Techno Jungang-daero, Hyeonpung-myeon, Dalseong-gun, Daegu, Korea, 42988. ^5^These authors contributed equally to this work.

*Corresponding author

Hyang-Sook Hoe, Ph.D.: Department of Neural Development and Disease, Korea Brain Research Institute (KBRI), 61 Cheomdan-ro, Dong-gu, Daegu, Korea, 41068

E-mail: [sookhoe72@kbri.re.kr](mailto:sookhoe72@kbri.re.kr)

Young-Man We, M.D., Ph.D.: Hyoo Medical Clinic Center, Teheran-ro, Gangnam-gu, Seoul, Korea, 06134

E-mail: [hyooclinic@naver.com](mailto:hyooclinic@naver.com)


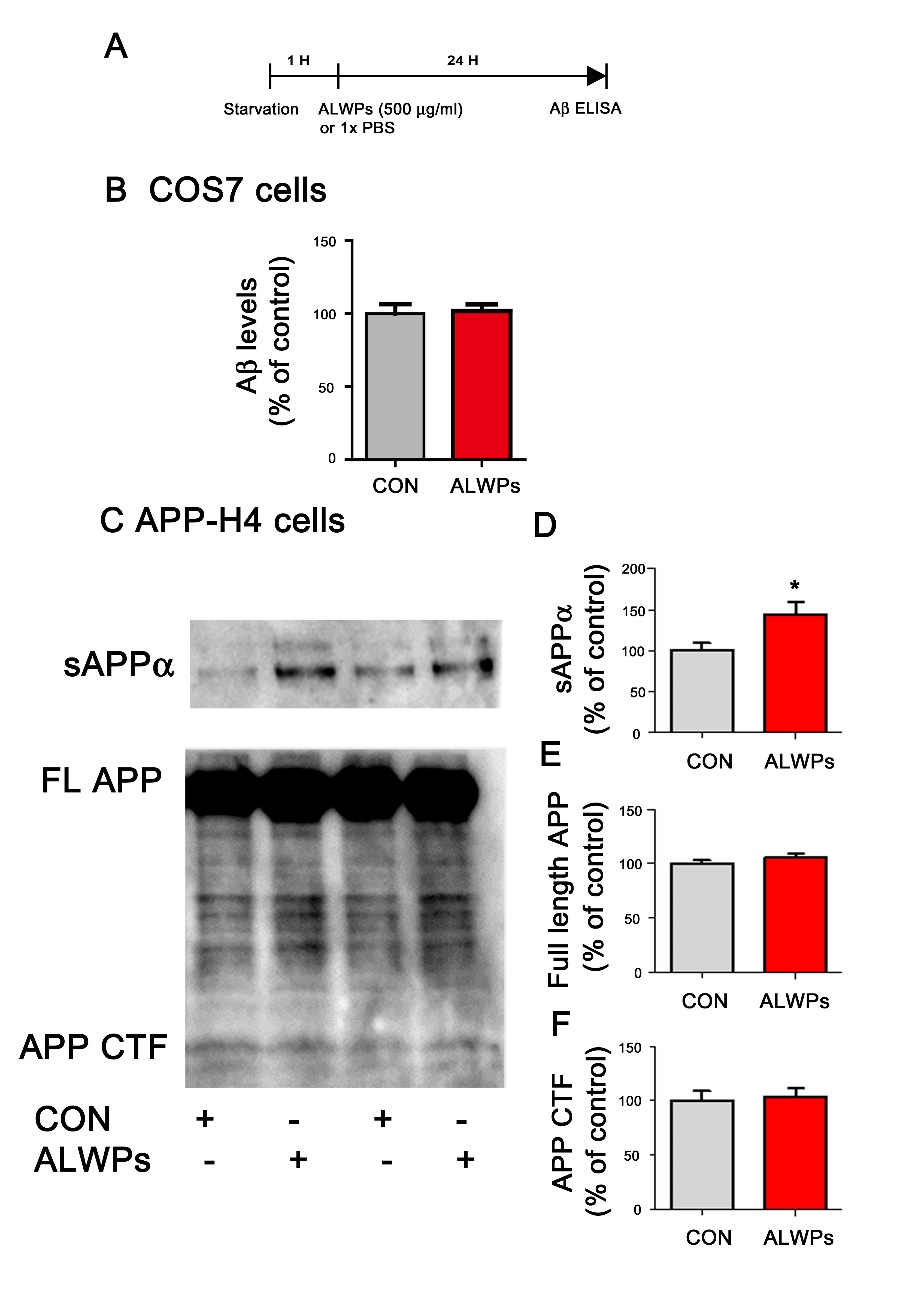


**Supplementary Figure. 1.** ALWPs significantly increase secreted APP alpha (sAPPα) levels in APP-H4 cells. (A-B) COS7 cells were transiently transfected with a construct expressing human APP for 24 hr and then treated with ALWPs (500 μg/ml) or PBS for 24 hr before performing Aβ ELISA (con, n = 16; ALWPs, n = 16). (C) APP-H4 cells were treated with ALWPs (500 μg/ml) or PBS for 24 hr, conditioned medium and cell lysates were collected, and western blotting was performed. (D-F) Quantification of data from C (con, n = 24; ALWPs, n = 24). **p* < 0.05.

**
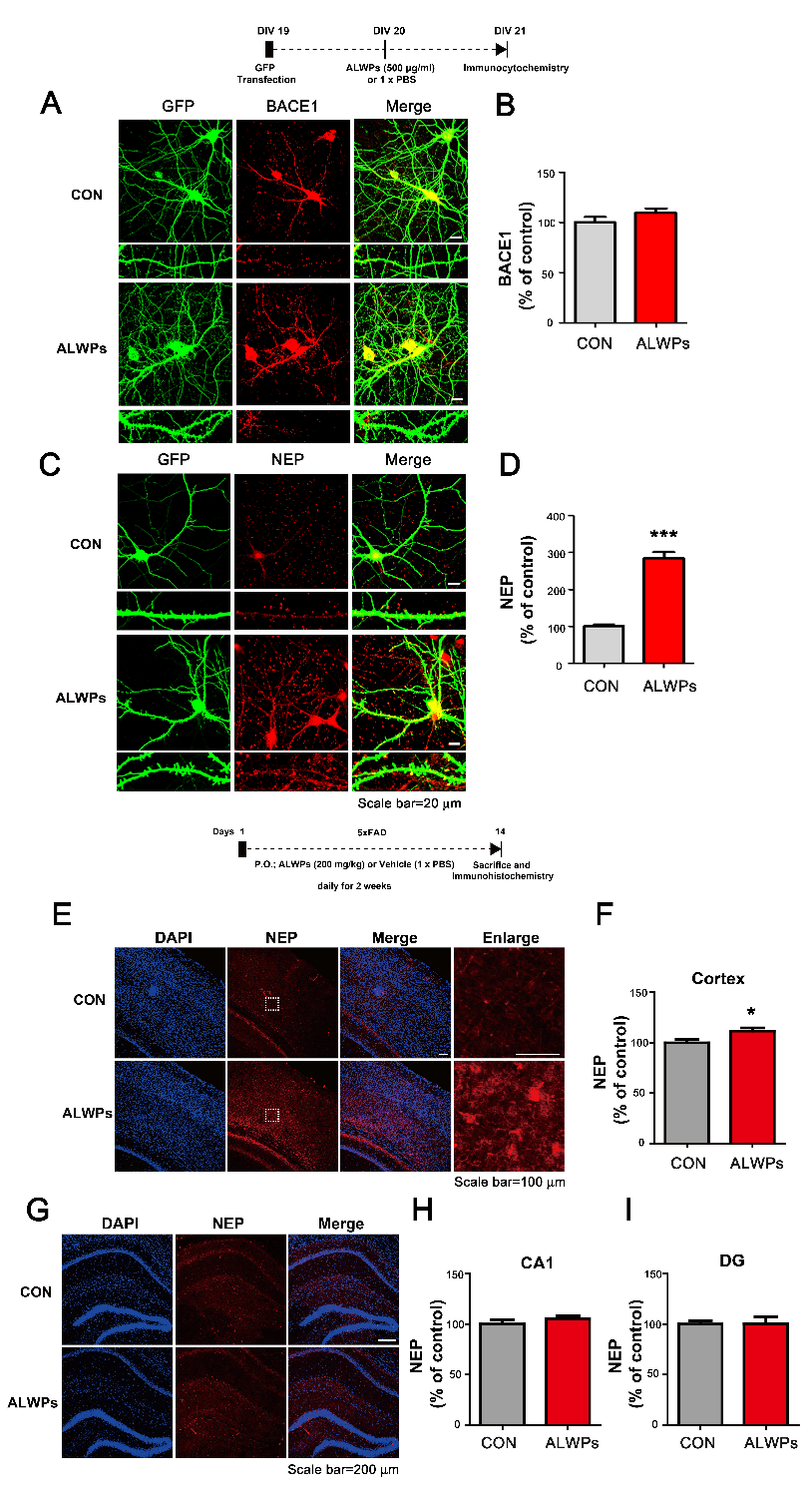
**

**Supplementary Figure. 2.** ALWPs do not alter BACE1 levels in primary hippocampal neurons. (**A**) Primary hippocampal neurons were transfected with GFP plasmid DNA for 24 hr, treated with ALWPs (500 μg/ml) or PBS for 24 hr, and immunostained with an anti-BACE1 antibody. (**B**) Quantification of data from A (con, n = 97 dendrites; ALWPs, n = 94 dendrites). (**C**) Primary hippocampal neurons were transfected with GFP plasmid DNA for 24 hr, treated with ALWPs (500 μg/ml) or PBS for 24 hr, and immunostained with an anti-NEP antibody. (**D**) Quantification of data from C (con, n = 90 dendrites; ALWPs, n = 90 dendrites). (**E**) 5x FAD mice were orally administered ALWPs (200 mg/kg, p.o.) or PBS daily for 2 weeks and immunostained with an anti-NEP antibody. Representative images of the cortex of 5x FAD mice are shown. (**F**) Quantification of data from E (con, n = 3 mice; ALWPs, n = 3 mice). (**G**) Representative images of the hippocampus of 5x FAD mice are shown. (**H**-**I**) Quantification of data from E (CA1 and DG; con, n = 3 mice; ALWPs, n = 3 mice). **p* < 0.05, **p* < 0.001.


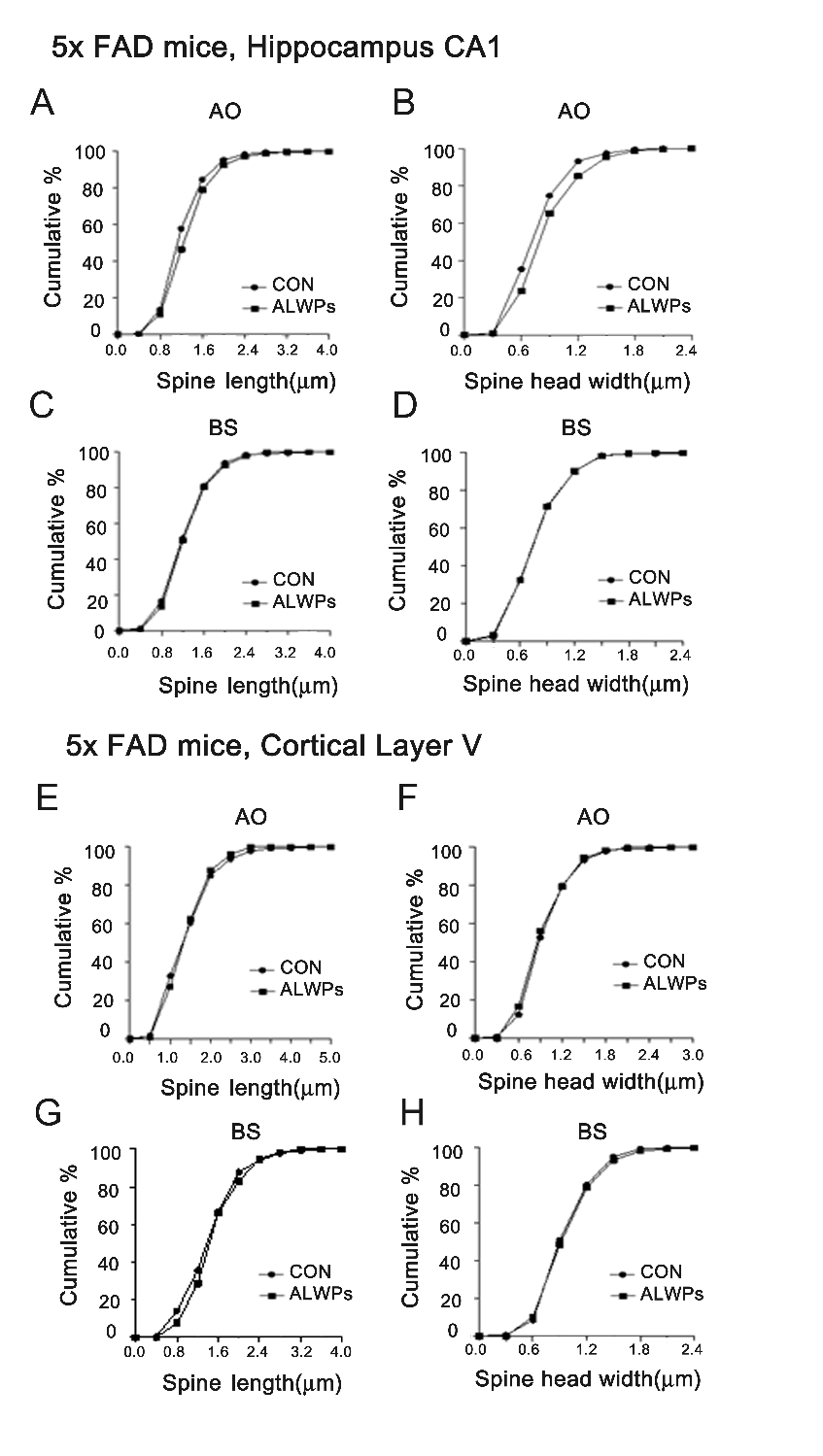


**Supplementary Figure 3.** ALWPs regulate dendritic spine morphology in hippocampus CA1 AO dendrites of 5x FAD mice. (**A**-**B**) The cumulative distribution percentage of spine length (A) and spine head width (B) in hippocampal CA1 AO dendrites of 5x FAD mice (n = 8 mice/group, Kolmogorov–Smirnov test). (**C**-**D**) The cumulative distribution percentage of spine length (C) and spine head width (D) in hippocampal CA1 BS dendrites of 5x FAD mice (n = 8 mice/group, Kolmogorov–Smirnov test). (**E**-**F**) The cumulative distribution percentage of spine length (E) and spine head width (F) in cortical layer V AO dendrites of 5x FAD mice (Kolmogorov–Smirnov test). (**G**-**H**) The cumulative distribution percentage of spine length (G) and spine head width (H) in cortical layer V BS dendrites of 5x FAD mice (n = 8 mice/group, Kolmogorov–Smirnov test).

**
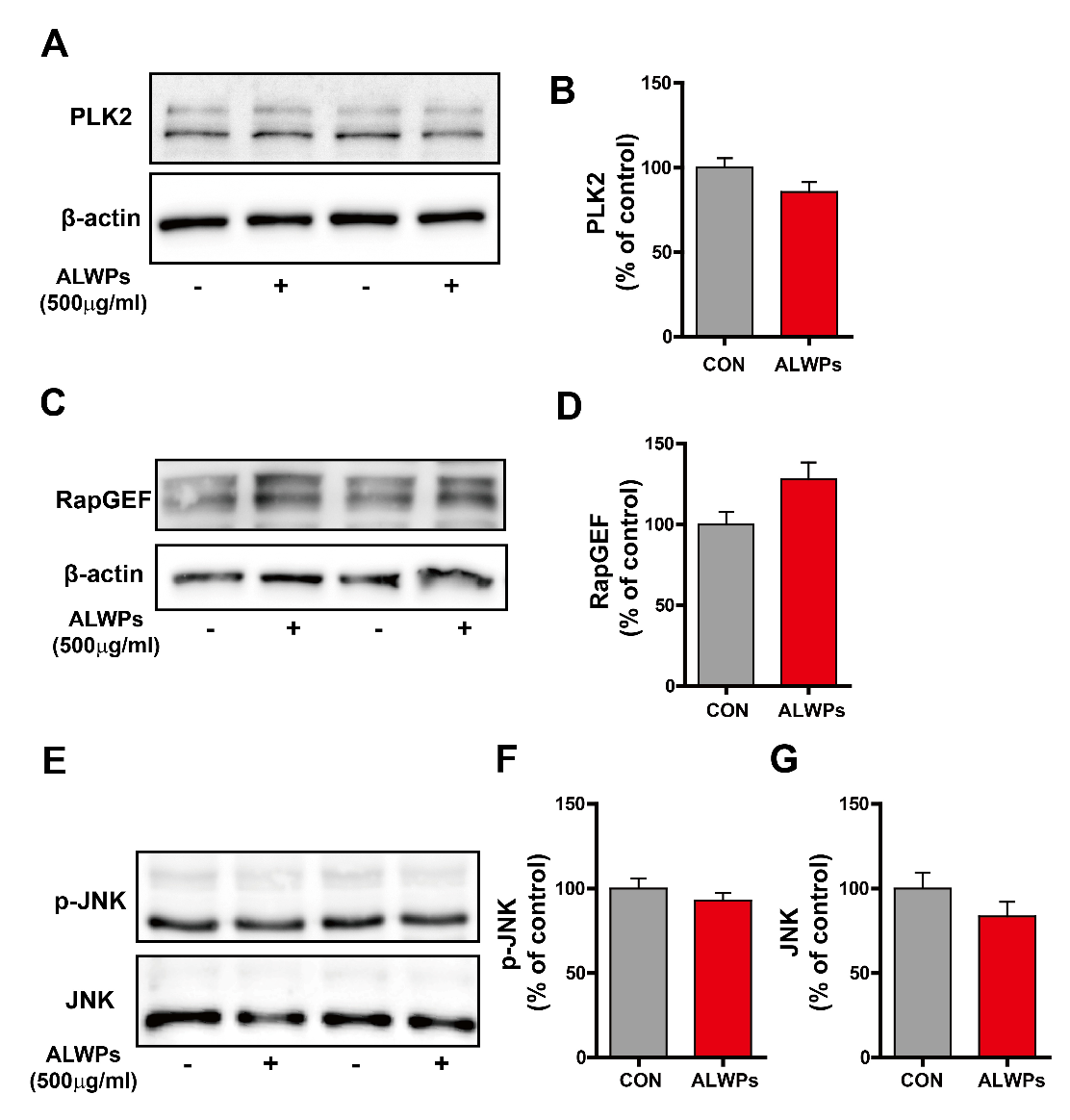
**

**Supplementary Figure 4.** ALWPs do not alter Rap signaling pathways. Primary cortical neurons were treated with ALWPs (500 μg/ml) or PBS for 24 hr and immunoblotted with anti-PLK2 (**A**-**B**), anti-RapGEF (**C**-**D**), and anti-p-JNK/JNK (**E**-**G**) antibodies (PLK2, n = 4; RapGEF, n = 4; p-JNK, n = 8; JNK, n = 4).
